# A pro-inflammatory diet is associated with an increased odds of periodontitis: finding from a case–control study

**DOI:** 10.1186/s40795-023-00760-7

**Published:** 2023-09-27

**Authors:** Reihaneh Sadat Ghaemmaghami, Mojtaba Bayani, Afrooz Nakhostin, Farhad Vahid

**Affiliations:** 1 Students Research Committee, Arak University of Medical Sciences, Arak, Iran; 2 Department of Periodontics, School of Dentistry, University of Medical Sciences, ArakArak, Iran; 3 Department of Restorative Dentistry, School of Dentistry, Arak University of Medical Sciences, Arak, Iran; 4 Nutrition and Health Research Group, Department of Precision Health, Luxembourg Institute of Health, Strassen, Luxembourg; 5 School of Health, Nutrition, and Health Research Group, Arak University of Medical Sciences, Arak, Iran

**Keywords:** Dietary Inflammatory Index (DII), Gum disease, Gingivitis, Food frequency questionnaire (FFQ), Periodontal diseases, Dental public health

## Abstract

**Objective:**

This study aimed to evaluate the inflammatory effect of diet using the dietary Inflammatory Index (DII) on the odds of periodontitis. We hypothesized that a diet with high DII scores (a pro-inflammatory diet) is associated with high chronic and systematic inflammation resulting in periodontitis.

Periodontitis is one of the most common inflammatory diseases that affect the tissues around the tooth and results from the interaction of bacterial infection and the host immune response. The DII shows the association between different food components and the level of specific inflammatory biomarkers.

**Method:**

The food intake of 87 cases with diagnosed periodontitis and 87 control was assessed using a 163-item valid food frequency questionnaire (FFQ). The DII was calculated based on the FFQ data. Logistic and linear regression models adjusting for multivariable confounders were used to investigate the odds ratio (OR) and 95% confidence intervals (CI) of developing periodontitis.

**Results:**

There was a significant difference between the mean intake of micronutrients and food groups, including saturated fatty acids (SFAs), iron, magnesium, manganese, vitamin C, crude fiber, selenium, chromium, whole fiber, caffeine, dairy, and meat, between patients with periodontitis and the control group (*p*-value˂0.05). DII scores in this study ranged from -3.13 to + 0.99. However, the periodontitis OR in the raw and multivariable-adjusted models was not statistically significant (multivariable-adjusted OR _tertiles 1 vs. tertiles 3_ = 2.00, 95%CI: 0.4–90.42, *p*-value = 0.08). A similar result was also observed in the continuous model of DII (multivariable-adjusted OR _DII continuous_ = 1.93, 95%CI: 0.30–98.79, *p*-value = 0.05).

**Conclusion:**

Although the OR was not statistically significant in crude models, a significant trend was found in multivariable-adjusted models. The results were promising since this is the first study to examine the association between diet-induced inflammation and dental disease. It is advisable to conduct additional studies with high sample sizes and other designs, such as prospective studies.

**Supplementary Information:**

The online version contains supplementary material available at 10.1186/s40795-023-00760-7.

## Introduction

Oral and dental diseases are part of the most critical public health problems. Due to pain, discomfort, and dysfunction, they can have devastating effects on people's lifestyles and have physical, social, and psychiatric consequences [1–3]. Periodontal disease is one of the most common chronic disorders that has plagued humans for centuries and is considered to be the dominant reason behind tooth loss in adults globally [4]. Severe periodontitis's global cost is estimated to be 54 billion dollars annually [5]. Periodontal disease is an inflammatory disease caused by the interaction of a bacterial infection and the host's immune response [6]. Epidemiological data show that in patients with periodontitis, serum levels of inflammatory biomarkers such as C-reactive protein (CRP) are increased compared to those without periodontitis [7]. In addition, inflammatory biomarkers such as interleukin (IL)-6, tumor necrosis factor (TNF)-α, matrix metalloproteinase-2 (MMP-2), and osteoclast accumulation are seen in tissues adjacent to periodontal pockets [8].

Risk factors such as smoking, diabetes, HIV/AIDS, family history, and certain medications increase the risk of having the mentioned disease, while contrarily, nutrition seems to contribute to preventing periodontal disease [9, 10]. Some studies investigated the association between periodontal disease and vitamin C, vitamin A, carotenoids, polyphenols, coenzyme Q, and the minerals such as iron, copper, and zinc [11]. Among the various antioxidants, there is more evidence for the usefulness of vitamin E and polyphenols [11]. Studies show that consuming certain nutrients, such as fermentable carbohydrates and SFAs, positively correlates to periodontitis risk [12]. Still, little research has been done on the effects of a general diet [12]. Woelber J et al., in a clinical trial study, reported that patients on a diet rich in omega-3 fatty acids and low in carbohydrates and animal proteins showed a significant reduction in gingivitis compared to controls. However, there were no intergroup differences in serological inflammatory parameters, serological omega fatty acids, and sub-gingival microbial composition [13]. A cohort study in the elderly Japanese population reported that a high intake of dietary antioxidants (vitamin C, vitamin E, alpha-carotene, and beta-carotene) was inversely related to the number of teeth involved in periodontal patients [14].

The Dietary Inflammatory Index (DII) indicates the relationship between different food components and the level of specific inflammatory biomarkers. These biomarkers include IL-10, IL-6, IL-1β, TNF-α, and CRP [15–18]. The primary purpose of using the DII is to evaluate the inflammatory potential of the diet based on its pro-inflammatory and anti-inflammatory properties and 45 different dietary parameters based on studies on cell, animal, and human cultures [16, 18]. These dietary parameters mainly include macronutrients, vitamins, minerals, flavonoids, and certain nutrients [19–21]. The data used to calculate the DII [15, 18, 22] can be obtained from various tools, such as the food frequency questionnaire (FFQ), 24-h dietary recall, and food records. Although existing studies have shown a link between certain food groups, such as antioxidants or omega-3 fatty acids, and periodontal disease, no specific research has been conducted on the association between pro-inflammatory and anti-inflammatory diet and periodontal disease. Whether the foods or nutrients are consumed together, dietary interactions or synergistic effects may alter the actual results of the nutrients under study. Therefore, the DII is designed to consider all nutrients regulating the inflammatory response. Most studies in Iran have focused on evaluating the relationship between nutrition and dental caries, and there are few studies on the relationship between food and periodontal disease [23]. This study aimed to investigate the association between dietary-induced inflammation and periodontitis. We hypothesized that a diet with high DII scores (an inflammatory diet) is associated with high chronic and systematic inflammation resulting in periodontitis.

## Methods

In this case–control study, the case group included people with periodontitis, and the control group included people without periodontitis. Two researchers examined individuals to determine whether or not they had periodontitis. According to William's probe approach, and performed based on the standard mirror in six-level (mesiobuccal, buccal, distobuccal, distolingual, lingual, and mesiolingual) of each tooth except the third molars. People with the following characteristics were considered a possible diagnosis of periodontitis and were referred to the periodontics department of the faculty. If the periodontist confirmed the periodontitis, the potentially diagnosed candidate would be included in the case group.

Detection of Clinical Attachment Loss (CAL) between teeth in at least two non-adjacent teeth, either as a periodontal envelope reported with a probe depth or as an analysis detected by observing the distance between the tooth CAL and the gingival margin. Detected CAL should not be of non-periodontal origins, such as 1) gingival resorption of trauma origin and 2) tooth decay that spreads to the cervical region of the tooth. 3) The presence of CAL in the distal region of the second molar and with malposition or removal of the third molar, 4) an Endodontic lesion that is drained through the marginal periodontium, and 5) the occurrence of vertical root fractures.

Other patients referred to the faculty identified in the examination without periodontitis were placed in the control group. After categorizing the candidates into “case” and “control” groups, the participants were guided by a brief explanation of how to respond to the FFQ questions. Participant names, age, height, weight, gender, menopause, smoking, diabetes, and educational status were recorded. The researchers then asked and recorded the frequency of consumption of each food item and the amount of consumption each time through two or three partial questions without direction (Example: How often do you use bread? How many portion sizes do you eat daily if the answer is daily?) The way they asked was neutral and without judging their eating habits.

### Inclusion criteria

- A) People over 18 years old who were referred to Arak Dental School to receive dental treatment
- B) They were willing to cooperate in the study
- C) People who did not have specific diets such as vegetarianism, weight loss, or obesity that resulted in significant weight loss during the year prior to the interview
- D) They did not have conditions such as pregnancy, lactation, neurological, liver, immune, kidney, or heart diseases

### Exclusion criteria

- A) Withdrawal from cooperation in answering the questions of the questionnaires
- B) People whose data analysis indicated receiving more than 5,500 kcal or less than 800 kcal per day (± 3SD)

### Sample size

According to the results of the study by Tomofuji et al., [24] the sample size was estimated to be 87 in case and control groups (α = 0.05, β = 0.2, the ratio of controls to cases = 1, percent of controls exposed = 8.2, odds ratio = 3.5).

$${\mathrm P}_0=\frac{\left(OR\right)P_1}{\left(OR\right)P_1+\left(1-P_1\right)}\;\mathrm{OR}=\frac{P_{1\left(1-P_0\right)}}{P_{0\left(1-P_1\right)}}$$

$${n}_{cases-Kelsey}=\frac{{\left({Z}_{a/2}+{Z}_{1-\upbeta }\right)}^{2}*p*\left(1-p\right)*\left(r+1\right)}{{r*\left({\mathrm{P}}_{0}-{\mathrm{P}}_{1}\right)}^{2}}$$

Total sample size = 174.

The protocol of the study was approved by the Faculty Research Council in Arak, Iran. It also was approved in the 335th meeting of the Arak University of Medical Science ethics committee, with the ethics code IR.ARAKMU.REC.1400.017.

Samples were selected from those referred to Arak Dental School, Arak, Iran. Before entering the study and examination, the research subject explained to them in simple language and the type of cooperation required, including being examined and answering the questionnaire. Written informed consent was obtained from all participants.

## Data collection

### Assessment of dietary intakes

The FFQ was used to collect dietary data. The FFQ records the frequency and amount of consumption of different foods in the past year. The FFQ is usually the most appropriate method of evaluating the diet in the long run. Easy to use, relatively low cost, and relatively fast estimation of people's typical diet, FFQ has become a practical tool [25, 26]. The questionnaire was based on Iranian food items and contained questions about the average frequency of consumption of food items according to the standard serving size or the amount that is usually more familiar to the community's people during the past year [25]. The FFQ includes legumes, meats, oils, rice, etc., and does not include mixed foods such as salads, soups, stews, etc., other than pizza. Individuals in the form of this questionnaire could report their answers by consumption times per day or week, or month or never. We used U.S. Department of Agriculture (USDA) servings such as a slice of bread, a medium apple, or a glass of milk for each portion of FFQ's food items, or otherwise household scales such as a tablespoon of beans or a spoonful of cooked rice [27].

Each food item's serving size (serving) was standardized based on grams. The Nutritionist IV software defined the code of each item along with the consumption gram [28]. The daily intake of each food item was calculated by multiplying the consumption frequency by the size of each food item. Seasonal food intake, including fruits, was estimated according to the number of seasons in which the food was available.

Then, the questionnaire's information was entered in grams, and after analysis, each person's average daily energy and nutrients intake was calculated. According to the exclusion criteria, individuals with a daily energy intake of more than 5,500 kcal or less than 800 kcal (± 3SD) were excluded from the study and replaced with new individuals (3 participants in the case and 7 in the control group).

The FFQ data were used to extract dietary items, including energy, carbohydrates, protein, total fat, alcohol, fiber, cholesterol, saturated fatty acids (SFA), monounsaturated fatty acids (MUFA), polyunsaturated fatty acids (PUFA), omega-3 and omega-6 fatty acids, vitamin A, vitamin B6, vitamin B12, vitamin C, vitamin D, vitamin E, Folic acid, magnesium, zinc, selenium, riboflavin, niacin, thiamine, iron, garlic, onion, tea, caffeine, and flavonoids; to calculate (estimate) the DII.

## Calculation of the DII

First, the global mean of each food parameter was subtracted from the actual intake of each food parameter. Then the result was divided by the global standard deviation, and z-scores were calculated.

$$Z\;=\;\frac{Global\;mean\;of\;the\;food\;item\;-\;Dietary\;intake\;of\;the\;food\;item}{Global\;SD}$$

Then this value was converted to the percentile score. Two minus one multiplied the centralized score for each food parameter. To obtain the DII score of each individual's diet, the percentile axis score of each dietary parameter was multiplied by the inflammatory effect score of the respective dietary parameter (inflammatory potential for each dietary parameter derived from the literature review). Finally, by summing all the DII scores related to the nutritional parameters, the total DII score for each participant was calculated. A higher DII score indicates a more "pro-inflammatory" diet and lower values indicate an anti-inflammatory diet [29, 30].

### Statistical analysis

First, DII tertiles were calculated using SPSS software, and individuals were divided into three groups based on DII tertiles. Then, to determine the association between DII tertiles and quantitative and qualitative variables, one-way analysis of variance (ANOVA) and chi-square tests were used, respectively.

We used multivariate logistic regression and continuous models (linear regression) to estimate the odds ratio (OR)s and 95% confidence interval (95%CI)s of periodontitis. We investigated the association between DII and periodontitis (continuous and tertiles) in raw models (without adjustments) and adjusted models (adjusted to demographic and lifestyle variables such as smoking, diabetes and age, sex, height, weight, body mass index (BMI), level of education, and menopause status). The significance of the OR for periodontitis in each diet DII was determined by considering the 95%CI.

## Results

DII scores in this study ranged from -3.13 to + 0.99. The mean and frequency of demographic characteristics and lifestyle of the case (*n* = 87) and control (*n* = 87) groups are given in Table 1. The age, BMI, smoking, diabetes, and education level in the case and control groups differed significantly (*p* < 0.05). The mean age, BMI, and percentage of smokers and diabetics in the case group were higher than in the control group. The level of education in the control group was higher than in the case group. Table 1 also compares the mean DII in the case and control groups. The difference between the mean DII in the case and control groups was not statistically significant. Supplementary Table 1 shows the demographic and lifestyle characteristics of DII tertiles. The ANOVA and chi-square tests did not show significant differences in DII tertiles' mentioned variables.

**Table 1:** General characteristics of people participating in the study based on case and control groups

Characteristics	Mean ± Standard deviation or N (%)		*P*-value*
	**Case ( *n* = 87)**	**Control (*n* = 87)**	
- Age (year)	$39.36\pm 12.05$	$44.60\pm 11.77$	**0.004**
- Height (m)	$1.68\pm 0.09$	$1.66\pm 0.10$	$0.172$
- Weight (kg)	$71.62\pm 11.79$	$74.97\pm 15.21$	$0.106$
- BMI (kg/m^2^)	$25.08\pm 3.60$	$26.77\pm 4.23$	$\bf 0.005$
- DII (continuous)	$-0.278\pm 0.75$	$-0.0939\pm 0.52$	0.062
DII (tertiles)			0.539
Third tertiles > 0.1	27 (32.1)	33 (37.9)	
Second tertiles -0.2 to 0.1	22 (25.2)	23 (26.4)	
First tertiles < -0.2	38 (43.6)	31 (35.6)	
- Gender			0.999
Female (%)	51 (58.6)	51 (58/6)	
Male (%)	36 (41.4)	36 (41.4)	
- Menopause (yes)	13 (27.5)	19 (37.3)	0.290
- Smoking (yes)	3 (3.4)	11 (12.6)	**0.026**
- Diabetes (yes)	2 (2.3)	9 (10.3)	**0.029**
- Educational status			**0.006**
Illiterate	0 (0.0)	2 (2.3)	
Diploma down	14 (16.1)	30 (34.5)	
Diploma & Diploma up	73 (83.9)	55 (63.2)	

^*^Independent sample t-test was used for comparing continuous variables, and a Chi-square test was used for categorical variables

*DII* Dietary Inflammatory Index, *BMI* Body Mass Index

Significant values are given in bold

However, there was a significant difference between the mean intake of eleven micronutrients, including SFA, iron, magnesium, manganese, vitamin C, crude fiber, selenium, chromium, total fiber, caffeine, and two food groups, including dairy and meat, between case and control groups (Table 2).

**Table 2:** Comparison of micronutrients and selected food groups in control and case groups

	**mean** $\pm$** standard deviation**		
**Variables**	**Controls**	**Cases**	*P*-value*
SFA (gr)	$34.68\pm 15.09$	$41.11\pm 19.60$	**0.016**
Iron (mg)	$26.82\pm 21.22$	$19.42\pm 9.67$	**0.004**
Magnesium (mg)	$435.89\pm 231.52$	$370.97\pm 141.11$	**0.027**
Manganese (mg)	$6.53\pm 4.38$	$5.42\pm 2.33$	**0.040**
Vitamin C (mg)	$282.36\pm 196.16$	$224.80\pm 106.95$	**0.016**
Selenium (mg)	$0.06\pm 0.023$	$0.07\pm 0.031$	**0.038**
Chromium (mg)	$0.26\pm 0.26$	$0.17\pm 0.116$	**0.033**
Total fiber (gr)	$34.44\pm 25.40$	$25.66\pm 12.55$	**0.004**
Crude fiber (gr)	16.37 $\pm$ 12.40	12.31 ± 6.02	**0.007**
Caffeine (mg)	$122.45\pm 88.37$	$163.17\pm 148.77$	**0.030**
Dairy (cup = 250ml)	$1.65\pm 1.21$	$1.24\pm 0.79$	**0.044**
Meat (oz. = 30g)	$5.30\pm 2.53$	$6.42\pm 3.35$	**0.014**

^*^Independent sample t-test was used for comparing variables. Significant values are given in bold

A comparison of the OR for periodontitis based on DII tertiles is given in Table 3. The first tertiles were considered the basis (reference), and then the chance of developing periodontitis in the second and third tertiles compared to the first tertiles is shown. The OR for periodontitis was non-significant in either the raw or adjusted models.

**Table 3:** Odds ratio and 95% confidence interval for the association between DII tertiles and periodontitis (*n*=174)

	OR in the DII tertiles (confidence intervals 95%)			*P*-value	
Models	Third tertiles >0.1	Second tertiles -0.2 to 0.1	First tertiles $\bf <-0.2$		
Raw model	1.49 (0.74 - 3.00)	1.28 (0.60 - 2.72)	Reference	0.51	0.25
Adjusted model^a^	2.00 (0.90 - 4.42)	1.20 (0.51 - 2.79)	Reference	0.66	0.08

^**a**^Adjusted for age, gender, smoking, diabetes, BMI, education level, and menopause

The OR for periodontitis based on linear (continuous) regression of the DII is given in Table 4. Although the smallest *p*-value was observed in the modified linear (continuous) regression model (*p* = 0.05), it was not statistically significant.

**Table 4:** Odds ratio and 95% confidence interval for the association between continuous DII and periodontitis (*n* = 174)

Models	OR in the DII (confidence intervals 95%)	*P*-value
Raw model	1.38 (0.76–2.53)	0.28
Adjusted model^a^	1.93 (0.98–3.79)	**0.05**

^a^Adjusted for age, gender, smoking, diabetes, BMI, education level, and menopause. Significant values are given in bold

## Discussion

This case–control study investigated the association between DII and periodontitis in adults referred to Arak Dental School, Arak, Iran. The present study showed that people with higher DII scores were more likely to develop periodontitis based on DII tertiles and the continuous variable, but the association was not statistically significant. The ORs for periodontitis based on linear and logistic regression of the DII were improved after adjusting for cofounders; however, the results were not statistically significant. The increased ORs could be indicative of the complex interplay between dietary choices, inflammation, and periodontal health. It is important to emphasize that association does not necessarily imply causation and further research is needed to establish a causal relationship. Additionally, the potential reverse causation should also be considered in the interpretation. It is possible that the progression of periodontitis itself might influence dietary habits, leading to altered food choices and DII scores. In addition, after adjusting for relevant confounding factors, the observed increase in the OR emphasizes the need for a comprehensive understanding of the underlying mechanisms contributing to the association between DII scores and periodontitis. Careful interpretation of the results, consideration of potential effect modifiers, and the investigation of causality through longitudinal studies will provide a more robust understanding of the relationship and inform effective preventive and therapeutic strategies for periodontitis. This is the first study to examine the association between DII scores and periodontitis in Iran. It is also the first case–control study to investigate this association. Previous studies measuring the relationship between DII and periodontal disease were population-based cross-sectional studies conducted in the United States [31, 32].

In the Li study [32], the association between periodontitis and energy-adjusted DII (E-DII) was significant in the continuous models. In the raw model, participants were 53% more likely to develop periodontitis in the highest E-DII than in the lower model and 2.66 times more likely in the modified model [32]. In addition, people in the third tertiles showed a higher prevalence of obesity, and their level of education was lower. In that study, DII scores (from -5.45 to 4.74) were broader than ours [32]. In addition, Machado's investigation concluded that there might be a link between DII and periodontitis because patients with a pro-inflammatory diet showed higher periodontal scales (mean probe depth and CAL) [31].

In a cross-sectional study in the United States, participants with the highest quartile of DII (pro-inflammatory diet) averaged more missing teeth after adjusting for confounders than those with the lowest quartile of DII (anti-inflammatory diet) [33]. These discrepancies may be due to differences in diet between Iran and the United States [34]. Many people in the United States and Europe eat Western diets [35]. These diets are mainly composed of meat, industrial foods, sugar, refined grains, alcohol, salt, and fructose, associated with reduced consumption of fruits and vegetables [36, 37] DII is a nutritional tool that reflects the levels of six inflammatory markers [16]. The inflammatory potential of different diets can be attributed to various nutrition components [16].

Several hypotheses have been proposed for biological interactions between inflammation and periodontal disease. Changes in inflammatory and immune responses, glucose intolerance, lipid profile abnormalities, changes in host immunity, increased macrophage activation, microvascular dysfunction, physiological responses to psychosocial stress, and pro-inflammatory secretion of adipose tissue, including TNF-a, IL-6 and CRP [38].

In the present study, the mean daily intake of SFAs and meat was significantly higher in the case group than in the control group. From a systemic perspective, a pre-inflammatory diet contributes to higher levels of systemic inflammation [39]. A pro-inflammatory diet is typically characterized by increased consumption of processed meats, red meats, saturated fats, and simple carbohydrates [40, 41]. The most common sources of SFAs include cakes, cookies, animal products, potato chips, popcorn, breakfast cereals, and candy [42].

In the present study, the average daily intake of crude fiber and total fiber in the control group was significantly higher than in the case group. The Nielsen population-based study showed an inverse relationship between dietary fiber intake and periodontal disease in adults over 30 years old in the United States [43]. Periodontal disease was associated with low consumption of whole grains but was not significantly associated with low consumption of fruits and vegetables [43]. The results of the Schwartz Longitudinal Study, which aimed to investigate the relationship between periodontal disease progression and fiber sources, showed that in men 65 years of age and older, only fruits with good to excellent fiber sources were associated witha lower risk of developing alveolar bone resorption and tooth loss. No significant association was observed in men under 65 [44].

Furthermore, the impact of weak gums on food choices in individuals with periodontitis warrants a more comprehensive analysis when interpreting the association between low dietary fiber intake and heightened periodontitis risk. The condition of weak gums may compel people to opt for softer and less fibrous foods, inadvertently leading to reduced consumption of fruits, vegetables, and high-fiber dietary options. Consequently, this dietary pattern could result in higher DII scores within the case group, potentially obscuring the true association between dietary fiber intake and periodontitis risk. Researchers and healthcare professionals must take into account this unique aspect of periodontitis patients' dietary choices and their potential influence on DII scores to arrive at a more nuanced understanding of the relationship between diet, inflammation, and oral health outcomes. By acknowledging the impact of weak gums on food preferences, future studies can delve deeper into the intricate interplay between diet and periodontal health, contributing to more tailored and effective approaches in managing periodontitis and promoting overall oral well-being.

A review study investigating the relationship between periodontitis and oxidative stress levels, antioxidants, including vitamin C and vitamin A, carotenoids, some polyphenols, coenzyme Q, and minerals iron, copper, and zinc, are compounds of antioxidant enzymes analyzed and among the various antioxidants, vitamin E and polyphenols had more evidence of beneficial effects [45]. Still, studies generally are not enough to rule out or determine which antioxidants are beneficial and which are not [45].

In the present study, the mean daily iron, chromium, and vitamin C intake was significantly higher in the control group. The association of selenium with periodontal disease has not been well studied. However, selenium is essential for immune responses, and serum levels are inversely related to inflammation and tissue destruction [46]. It has also been reported that low serum selenium levels may be associated with the severity of periodontal disease [47]. Although studies are limited, maintaining selenium levels in periodontal disease may help manage them [48]. Nevertheless, the mean daily selenium intake was higher in the case group in the present study.

An important strength of this study is that it is the first study in Iran to examine periodontitis as a DII-related outcome. In addition, the present study calculated the DII based on the FFQ consumption in one year, while other studies evaluated diet-related inflammation based on 24-h diet recalls. Another important strength of the present study is using a valid and reproducible FFQ that comprehensively assesses the primary sources of nutrients in the diet. However, there may be some inherent measurement bias in the FFQ. The limitation of the one-meal/nutrient approach is that foods or nutrients are usually consumed with other foods and nutrients. Thus, food interactions may alter the actual effects of the food or nutrient being studied. In formulating DII, a different approach was taken, focusing on the functional impact of foods and nutrients. DII tertiles on reviews and ratings of articles reviewed on diet and inflammation. It also standardizes people's dietary intake of pro-and anti-inflammatory food items with global reference values, resulting in values ​​that are not unit-dependent and can be used for comparison in studies [16]. This study faced limitations that could be considered in future studies. The relatively small sample size can be mentioned as one of the limitations of this research.

On the other hand, as a limitation, some factors related to periodontitis, such as the oral health status of the participants in this study, were not examined. Participants in this study were limited to a public dental center's clients, which may have influenced the results. Another limitation of this study, as in other case–control studies, was the presence of recall bias and selection bias.

## Conclusion

The present study showed that the difference in periodontitis's ORs was non-significant in either the raw model or the adjusted model in the DII tertiles. Although the OR was not statistically significant in crude models, a significant trend was found in multivariable-adjusted models. It is suggested that future studies be conducted with a larger sample size and on participants referred to various centers at the regional/national levels. In addition, participants' oral health status should be considered a variable related to periodontitis. However, there was a significant difference between the mean intake of micronutrients and food groups, including SFA, iron, magnesium, manganese, vitamin C, crude fiber, selenium, chromium, whole fiber, and caffeine, dairy, and meat group between patients with periodontitis and the control group. Therefore, informing and educating nutritionists on these patients to prevent possible nutritional deficiencies, avoid unhealthy nutrition, and improve their overall health seems necessary.

### Supplementary Information

**Additional file 1: Supplementary table 1.** General characteristics of people participating in the study based on the level of the dietary inflammatory index (DII).

## Data Availability

The data presented in this study are available on request from the corresponding Author.
