# Supplementary material for: A pro-inflammatory diet is associated with an increased odds of periodontitis: finding from a case–control study
Source: BMC Nutr. 2023 Sep 27;9:109. doi: 10.1186/s40795-023-00760-7 (PMC10537435; doi:10.1186/s40795-023-00760-7)
Supplement: Supplementary file 1 — Additional file 1: Supplementary table 1. General characteristics of people participating in the study based on the level of the dietary inflammatory index (DII). [file 40795_2023_760_MOESM1_ESM.docx]

**Supplementary materials**

| **Supplementary table 1.** General characteristics of people participating in the study based on the level of the dietary inflammatory index (DII). | | | | |
| --- | --- | --- | --- | --- |
| **P-value*** | **First tertile**  $\mathbf{<}\mathbf{-0.2}$ | **Second tertile**  $\mathbf{-0.2}\boldsymbol{to}\mathbf{0.1}$ | **Third tertile**  $\mathbf{>0.1}$ | **Characteristics** |
| 0.102 | $43.72\pm13.41$ | $42.32\pm11.07$ | $38.72\pm11.17$ | Age (year) |
| 0.698 | $1.67\pm0.09$ | $1.68\pm0.11$ | $1.68\pm0.09$ | Height (m) |
| 0.971 | $73.60\pm12.95$ | $73.04\pm14.53$ | $73.16\pm13.85$ | Weight (kg) |
| 0.554 | $26.34\pm4.10$ | $25.66\pm4.07$ | $25.66\pm3.78$ | BMI (kg/m2) |
| 0.355 |  |  |  | Gender |
|  | 45 (65.2) | 34 (54.8) | 23 (58.6) | Female (%) |
|  | 24 (34.8) | 28 (45.2) | 20 (46.5) | Male (%) |
| 0.349 | 18 (26.1) | 10 (16.1) | 5 (11.6) | Menopause (yes) |
| 0.447 | 5 (7.2) | 7 (11.3) | 2 (4.7) | Smoking (yes) |
| 0.689 | 5 (7.2) | 2 (3.2) | 4 (9.3) | Diabetes (yes) |
| 0.494 |  |  |  | Educational status |
|  | 0 (0.0) | 1 (1.6) | 1 (2.3) | Illiterate |
|  | 7 (24.6) | 19 (30.6) | 8 (25.3) | Diploma down |
|  | 34 (75.4) | 42 (67.7) | 34 (75.4) | Diploma & Diploma up |
| * One-way ANOVA was used for comparing continuous variables, and the Kruskal-Wallis test was used for categorical variables. | | | | |
